# Evaluation of Chitosan-Based Axiostat as Hemostatic Dressing for Endovascular Procedures in Patients with Leriche Syndrome on Anticoagulant Therapy

**DOI:** 10.3390/ph18040584

**Published:** 2025-04-16

**Authors:** Paolo Perri, Federica Curcio, Michele De Luca, Paolo Piro, Sonia Trombino, Roberta Cassano

**Affiliations:** 1 Department of Vascular and Endovascular Surgery, Annunziata Hospital, 1 Via Migliori, 87100 Cosenza, Italy; p.perri@aocs.it (P.P.); p.piro@aocs.it (P.P.); 2 Department of Pharmacy, Health and Nutritional Science, University of Calabria, Arcavacata, 87036 Rende, Italy; federica.curcio@unical.it (F.C.); michele.deluca@unical.it (M.D.L.)

**Keywords:** hemostatic dressing, chitosan, axillar access site, manual compression, bleeding

## Abstract

**Background/Objectives:** The safe completion of a non-invasive procedure is crucial to the success of an endovascular approach. Chitosan, a natural polysaccharide derived from chitin, is an ideal material for the study and application of medical devices in post-operative wound management. **Methods:** The present work is based on a retrospective study conducted on a sample of patients treated with Axiostat (a sterile, single-use, non-absorbable dressing), composed of 100% chitosan and designed to instantly stop bleeding through a mucus adhesion mechanism for the treatment of conditions such as Leriche’s syndrome. The objective was to evaluate the efficacy and safety of the hemostatic Axiostat dressing in patients undergoing anticoagulant and/or antiplatelet therapy in whom endovascular procedures using the axillary artery as an access site are performed to treat Leriche syndrome. **Results:** The obtained results showed that Axiostat is safe and effective in promoting hemostasis at the axillary vascular access site even when prolonged hemostasis was required in patients on antiplatelet and anticoagulant therapy. The mean time to hemostasis was 5.75 min in all types of patients considered.

## 1. Introduction

Leriche syndrome is a condition characterized by reduced blood flow in the arteries that carry blood to the lower extremities [1,2]. It is considered a form of aorto-iliac occlusive disease that requires effective hemostasis after endovascular procedures. In fact, the treatment of Leriche syndrome consists of performing aorto-bifemoral bypass with a 5-year patency rate of 85% to 94% but a mortality rate of 3.3% to 4.6% [3,4].

With recent advances in catheter technology and angiographic technique, there has been a dramatic shift toward an endovascular approach with a high technical success rate and low morbidity [5,6], but with limitations still related to achieving hemostasis of the vascular access site. In this regard, traditional methods, such as manual compression (MC) and vascular closure devices (VCDs), have limitations, including prolonged hemostasis time and high cost. Chitosan-based hemostatic dressings, such as Axiostat, offer a potential solution due to their rapid coagulation mechanism and biocompatibility [7]. In this regard, functional chitosan-based hemostatic dressings play a key role. They possess characteristics such as the ability to stop bleeding within two minutes, adequate biodegradability and bioabsorbability, clinical safety, ease of use and low cost [8,9]. Often, these properties are attributable to the polymer they are made mode, such as chitosan. The latter is considered the second most abundant polysaccharide, consisting of glucosamine and N-acetylglucosamine linked by β-(1–4) glycosidic bonds. It is a widely used biomaterial as it has numerous advantages, including immunogenic to human tissues and cells, does not cause biological rejection, and does not cause allergic reactions. It is highly degradable as it tends to be hydrolyzed into glucosamines that can be absorbed by the body or excreted through metabolism without accumulation in the body. It is a polymer with antibacterial properties and high hemostatic power.

**Figure 1:**
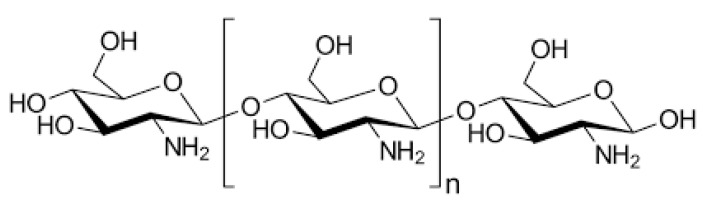
Chitosan structure.

The hemostatic action of chitosan can be attributed to its cationic form (Figure 1); it is positively charged in a neutral, acidic environment and is likely to attach to negatively charged red blood cell membranes through electrostatic interactions, promoting red blood cell coagulation and increasing platelet adhesion to achieve hemostasis. It is also able to promote wound healing through a mechanism in which the release of N-acetyl-β-D-amino glucose triggers the proliferation of fibroblasts, while chitosan monomers can also accelerate the orderly deposition of collagen and stimulate the increase in natural hyaluronic acid synthesis at the wound site [10,11].

**Scheme 1:**
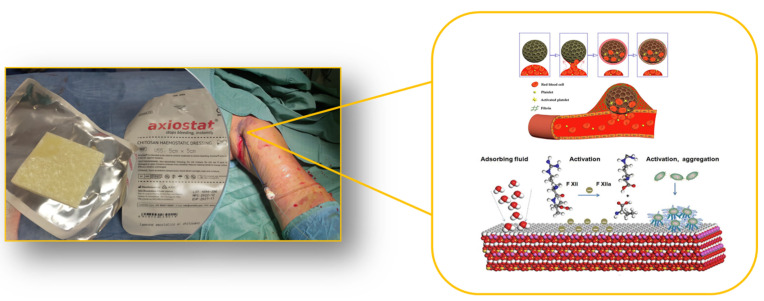
Axiostat application on axillar access in Leriche syndrome treatment.

In this work we will consider Axiostat, a sterile, single-use, non-absorbable and FDA-approved 93/42/EEC medical device dressing consisting of a highly porous chitosan matrix with a honeycomb microstructure, capable of promoting the formation of a mechanical barrier at the bleeding site that leads to the formation of fibrin, creating a plug that extends across the wound site, thus stopping the bleeding. This study aims to evaluate the efficacy and safety of the Axiostat hemostatic dressing in promoting hemostasis by manual compression of the axillar arterial access site in patients with Leriche syndrome undergoing anticoagulant and/or antiplatelet therapy, in whom axillary access is preferred for endovascular procedures (Scheme 1).

## 2. Results

### 2.1. Population Study

During the study interval (September 2022–February 2024), 60 patients underwent closure with manual compression of the left axillary arterial access site with the aid of the hemostatic dressing Axiostat. The mean age was 67.4 years, and 50.8% had a mean body mass index value of 27 (Table 1).

### 2.2. Procedure Data

The most frequently used size of the vascular introducer sheath was the 6 Fr and/or 8 Fr, with an average number of punctures of the vascular access site per patient of 1.2 (±0.2). The number of punctures was calculated considering each time the needle was removed from the skin after its insertion. Patients undergoing the vascular procedure continued the drug regimen they were already on before the procedure.

A total of 15 patients were on antiplatelet therapy (25%), 9 were on anticoagulant therapy (15%), 3 were on antiplatelet and anticoagulant therapy (5%) and 33 were on dual antiplatelet therapy (55%), as shown in Table 2. The mean INR value, measured before and after treatment, was 1.3 (±0.2), confirming the fact that in this type of procedure, it is not necessary to suspend the patients’ anticoagulant regimens as the immediate hemostatic action of Axiostat reduces local hemorrhagic complications, ensuring that therapeutic INR levels are maintained.

### 2.3. Outcomes E Follow-Up

The primary success rate resulting from the application of Axiostat was 100% in patients undergoing endovascular treatment undergoing single-antiplatelet and double-antiplatelet therapy; whereas it was 95–98% in patients undergoing anticoagulant and/or combined anticoagulant therapy in whom prolonged blood clotting increased the Axiostat-induced hemostasis time by a few minutes (Table 3).

**Figure 2:**
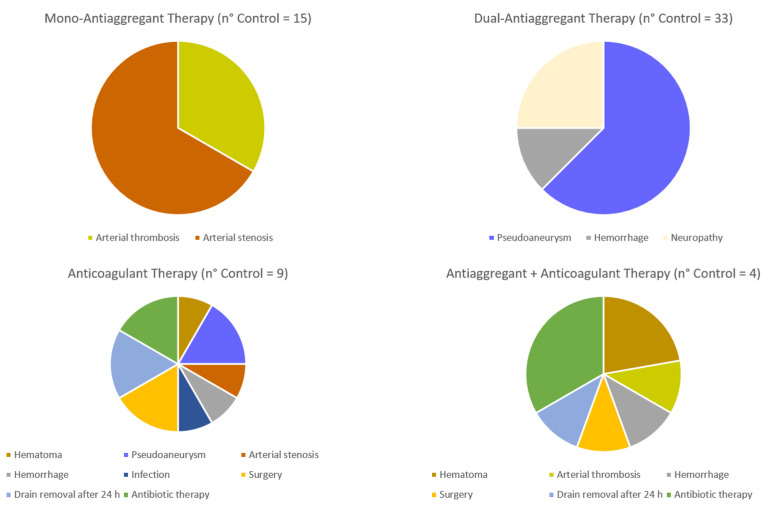
Graphical representation of the distribution of complications.

The rate of access-site complications was 1.8%, related to one case of hematoma in patients receiving anticoagulant therapy and two cases in patients receiving anticoagulant and/or combination antiplatelet therapy. There were also five cases of pseudoaneurysm formation in patients on dual antiplatelet therapy and two cases in those on anticoagulant therapy. In addition, only 1.2% required surgical intervention with closure of the axillary artery due to excessive bleeding and/or infection of the access site (Table 4). There were no cases of dissection or arteriovenous fistula formation. Healing with follow-up of the axillary access site at 7 and 30 days occurred in 99.9% of cases (Figure 2). No patients experienced any complications in either procedure.

### 2.4. Hemostasis Evaluation

**Figure 3:**
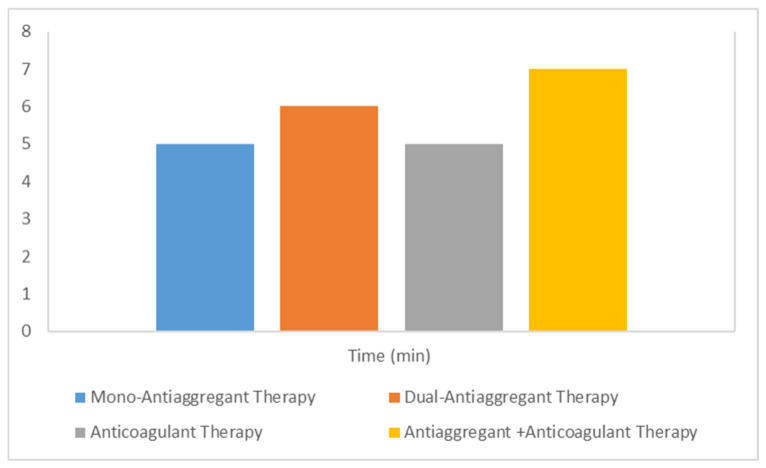
Graphical representation to evaluate the hemostasis time achievement of different groups of patients.

At the end of the 10 min observation period, the hemostatic success of lesions treated with Axiostat was 99.5%. In most cases of failure, blood leaked from under a contiguous gel mass or occurred in patients using antiplatelet and anticoagulant therapy (not discontinued prior to surgery). Controlling for differences in the level of pre-treatment bleeding, the median time for hemostasis was approximately 5 min after its use combined with manual compression by the surgeon (Figure 3). At the end of the 10 min observation period, the hemostatic success of lesions treated with Axiostat was 100% (Table 5).

## 3. Discussion

In surgery, a functional hemostatic dressing should be able to rapidly stop bleeding and ensure high biodegradability, bioabsorbability and adequate clinical safety. In addition, unlike conventional cotton gauze, a functional dressing should be easy to use, low cost and ensure firm adhesion to the surface of damaged tissue, with the ability to rapidly induce hemostasis, even in patients who are taking anticoagulants or are coagulopathic. In this monocentric retrospective study, we focused our attention on the application and efficacy of the chitosan-based Axiostat dressing in promoting hemostasis by manual compression of the arterial access site in patients with Leriche syndrome already on anticoagulants and/or antiaggregants, in whom axillary access was preferred for the endovascular procedure. There are no guidelines in the literature that condition the use of an innovative closure system (VCD) over traditional ones with manual compression (MC) after percutaneous access [12]. The choice of closure technique at the endovascular access site and the type of dressing to be used depends on the skill of the operator, the number of accesses performed by the operator on that artery, the number of procedures performed at that center, arterial access, and the availability of closure devices available at that time in the hospital. In this regard, it is useful to specify that alginate dressings could also be considered innovative dressings for the closure of vascular access sites. They are, in fact, like chitosan dressings, highly biocompatible and biodegradable and initiate the coagulation process by contact activation (thrombin converts fibrinogen to fibrin fiber), leading to the formation of blood clots. However, the natural hemostatic power of alginate dressings can be increased by exploiting the chemical nature of the polymer with its many functional groups that can be derivatized with other substances or combined with other natural or synthetic polymers.

The results obtained from our studies show us that the primary, secondary and clinical technical success rate was 99.9%, confirming Axiostat to be safe and effective in promoting hemostasis of the axillary vascular access site even when prolonged hemostasis was required. The mean hemostasis time was 5.75 min in all patient types considered. In patients on dual antiplatelet therapy and with anticoagulant and antiplatelet administered together, the hemostasis time was found to be longer due to the increased blood flow, which does not allow easy platelet plug formation. However, this does not constitute an obstacle for a surgeon who does not consider it appropriate to discontinue the administration of these drugs as they do not affect the ionic mechanism of chitosan. Underlying these positive values is, above all, the ability of this chitosan-based dressing to activate a hemostatic process by exploiting the chemical/physical characteristics of the polymer. Importantly, most biological and chemical applications of chitosan, especially in terms of hemostasis, are based on its cationic properties and its versatility as a biomaterial [13]. Specifically, by coming into contact with negatively charged platelets and erythrocytes, chitosan is able to absorb water and convert it into an adhesive (gel-like) element that adheres to the damaged tissue. This process is particularly effective for bleeding wounds under opposing pressure. The gauze’s high water absorption slows the blood flow, making it denser [14]. In fact, the hemostatic process induced by Axiostat exploits the attractive Coulomb force, i.e., the H+ ions continuously released by the chitosan into the blood reduce the negative charges on the surrounding red blood cells and thus the repulsive electrical force of the double layer between the red blood cells, which end up adhering to the polymer, forming a mucoadhesive barrier that promotes coagulation [15,16]. In the case of Axiostat, the microscopic porous structure of the dressing provides a unique molecular chemistry to the chitosan that prolongs its positive charge even under physiological conditions. Indeed, when Axiostat is pressed against damaged tissue, it creates a space within its pores that leads to mechanical interlocking with the tissue surface, providing instantaneous bioadhesion and resulting in the diffusion of surface-bound molecules (red blood cells and platelets) into the pores themselves [17,18]. In vivo applications of Axiostat have revealed its excellent hemostatic properties by minimizing or avoiding serious complications such as hematoma, recurrent hemorrhage, pseudoaneurysms, vessel thrombosis, infection and wound dehiscence. Dressing-induced hemostasis can be assessed directly by the surgeon recording hemostasis time, blood loss and hematoma formation after the application of the chitosan dressing and indirectly by assessing bleeding-related factors, which can serve as surrogate markers (e.g., aptt and platelet count). In patients on a regimen of antiplatelet and/or anticoagulant therapy, indirect clinical evaluation is not recommended because values may be altered by drug therapy. Specifically, in the surgical cases we analyzed, it was observed that only in a small percentage of patients (1.2%) did complications involving hematoma formation occur, following an increase in systolic blood pressure that forced the operator to prolong manual compression, replacing the Axiostat dressing several times; among the observed complications, pseudoaneurysms were more common in patients undergoing multiple punctures. This suggests that operator technique may influence outcomes and underscores the need to standardize puncture protocols. In our retrospective analysis, we observed that although the number of samples we analyzed was higher than what is normally reported in the literature for this type of treatment, the lack of randomization and the absence of a control group might account for some of the limitations of the investigation [19,20].

**Figure 4:**
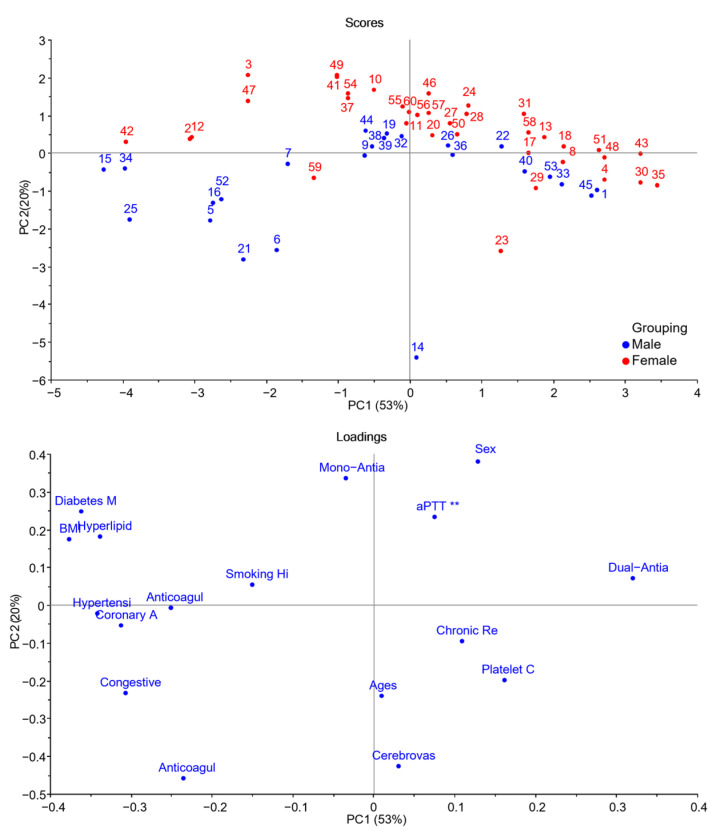
Scores and loading plots calculated in chemometric analysis, PC1 vs. PC2. ** activated partial thromboplastin time.

The present study had several limitations, such as those associated with comparison with similar studies performed in other vascular surgery departments of different hospitals to better understand aspects, such as efficacy and safety, associated with the use of chitosan-based dressings in the treatment of vascular access sites in patients undergoing endovascular procedures for Leriche syndrome. Since our retrospective single-center observational study is retrospective, it would need to be extended, with the same criteria and procedure, to other vascular surgery departments and especially to larger patient populations. In addition, considering that CT scans in the present study are not performed at the last follow-up, due to the absence of symptoms in the patients, one could consider including them while avoiding neglecting aspects that could be crucial to the reliability of the investigation performed. Finally, while having clearly defined inclusion and exclusion criteria, it would be useful to design a detailed protocol with standardized and cross-checked data collection forms, matching patients with cross-checks aimed at minimizing bias as much as possible. In addition, a pilot trial could be conducted to test the forms and coding methods. In the study on the evaluation of antibacterial activity conducted by Kumar et al., a 5 cm Axiostat dressing was used as a sample, while sterile cotton gauze of a similar size served as a control. The results showed that Axiostat has the ability to act as a barrier against bacteria, such as *S. aureus*, *S. epidermidis*, *Micrococcus luteus*, *Pseudomonas eruginosa*, *Escherichia coli* and *Salmonella abony*, and prevent their entry through its surface [21]. This activity is said to be linked to chitosan’s ability to bind to the negatively charged cell walls of microorganisms at the site of penetration, inhibiting the synthesis of mRNA and proteins through penetration into the nucleus, resulting in cellular osmosis of the microorganisms [22,23]. All of these could reduce the frequency of administration of antibiotic therapy and the occurrence of infections at the endovascular access site. No adverse reactions related to the occurrence of allergic reactions from in vitro tests and in vivo application of Axiostat have been reported in the literature, as also demonstrated by the clinical cases we analyzed. The product we use is accompanied by a scientific compendium detailing cytotoxicity, skin sensitization, acute systemic toxicity and intracutaneous reactivity tests. Furthermore, being a CE-certified device issued under the Medical Device Directive 93/42/EEC, it undergoes gamma sterilization procedures performed prior to marketing. At discharge and at 7- and 30-day follow-up, performed in the outpatient clinic, no patients reported complications at the endovascular access site treated with Axiostat [24]. A PCA of the study data, using the first two principal components (PCs), captured approximately 73% of the total variance. Figure 4 illustrates the projection of the samples studied and their describing variables onto the new PCs calculated by PCA. The distribution of the samples on the score plot and the variables on the loading plot highlights the correlation between key patient characteristics (such as BMI, diabetes, hypertension or smoking habits) and hemostatic outcomes. Patients with metabolic disorders tended to exhibit prolonged bleeding times. By examining the score and loading plots, which project samples and variables relative to the principal components, it was evident that risk factors like diabetes mellitus, hyperlipidemia and high BMI predispose patients with Leriche syndrome to advanced coronary artery disease, often necessitating treatment with antiplatelet and/or anticoagulant therapy. Another significant observation was the association between hypertension and smoking, particularly in female patients, who had a higher incidence of the disease in the presence of these factors. In addition, a higher incidence of Leriche’s syndrome was observed in male patients, where the presence and correlation of multiple risk factors significantly influenced the onset of the disease. In diabetic patients, prolonged hemostasis time may be attributed to impaired platelet function and vascular integrity. Future studies should explore tailored approaches for this subgroup. The presence and correlation of risk factors on the incidence of Leriche syndrome are closely related to the results derived from hemostasis induced by a good closure device since, for example, in patients with metabolic disorders such as diabetes or a high BMI, a greater tendency toward prolonged bleeding times is inferred. In addition, patients who develop Leriche as a result of a previous history of smoking and hypertension are more prone to the formation of fibrocalcific plaques that can create stenosis or even arterial occlusions from the aorta to the iliac-femoral bifurcations. In this type of patient, the use of mechanical closure systems (prolene knots, resorbable sutures, etc.) to treat the vascular access site may further predispose the patient to stenosis formation or the occlusion of the arterial lumen by incorporating a plaque into the suture itself. Applying state-of-the-art dressings such as Axiostat not only ensures immediate hemostasis but also avoids the process described above and possibly ensures the reuse of the same vascular access site to intervene if needed, given the chronic-degenerative nature of atherosclerotic pathology. Future research directions should be clearer.

## 4. Materials and Methods

### 4.1. Study Design

This study is a monocentric analysis conducted at the Annunziata Hospital in Cosenza, Italy, in collaboration with the Department of Pharmacy, University of Calabria, of data collected from consecutive patients who underwent, from September 2022 to February 2024, closure of the left axillary artery using the Axiostat hemostatic dressing. Axiostat was purchased from Axio Biosolutions Pvt. Ltd. (Gujarat, India). Inclusion criteria were (I) endovascular treatment with sheath placement in the left axillary artery introducer and sheath size between 4 Fr and 8 Fr; (II) age greater than 18 years; (III) left axillary artery size greater than 6 mm; (IV) the presence or absence of fibrocalcific plaques; (V) the presence or absence of course abnormalities at the femoral bifurcation; (VI) and surgeon’s decision to perform manual compressive hemostasis of the vascular access site. Exclusion criteria were (I) age less than 18 years; (II) previous surgery in the access area and/or previous placement of VCD at the access site; (III) previous endovascular treatment or vascular access or use of catheters at the vascular access site; (IV) risk of excessive bleeding recommendations of the CIRSE standards of practice on perioperative anticoagulation; (V) and pregnancy.

### 4.2. Data Collection and Procedure

**Figure 5:**
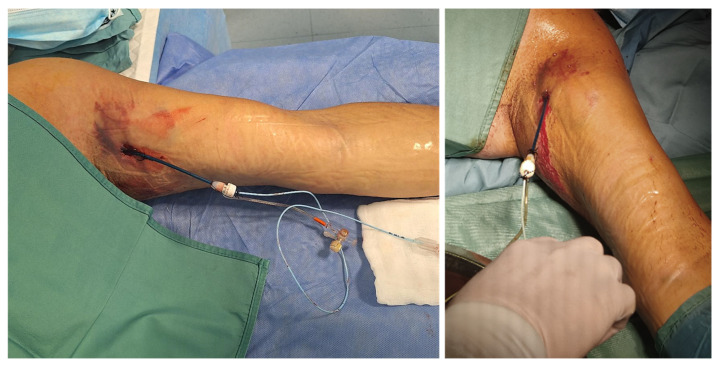
Introduction of the sheath 8 Fr for the endovascular procedure.

Patients undergoing an endovascular angioplasty procedure, in which axillary access is preferred for the treatment of Leriche syndrome at the level of the aorto-iliac tract, undergo local anesthesia with lidocaine by injection through the skin and subcutaneous tissue to the perivascular fascia. Subsequently, the placement of the introducer sheath is assessed with a CT study and an ultrasound analysis (B-Mode ultrasound, LINEAR 7 mHZ probe, SONOSITE M-TURBO ultrasound, Bothell, WA, USA) of the axillary artery in the section between the axillary cord and the brachial artery. A percutaneous access is then performed using a hollow trocar needle, preferably to the proximal third of the artery. Using Seldinger’s technique [25], the guide wire and rod are inserted under fluoroscopic control, with an initial flush with saline solution and administration of 1 cc of heparin (5000 I.U.) Figure 5. At this point, a metal stent (Begraft 8 × 57 mm) is placed in kissing stent mode at the level of the iliac aortic tract.

**Figure 6:**
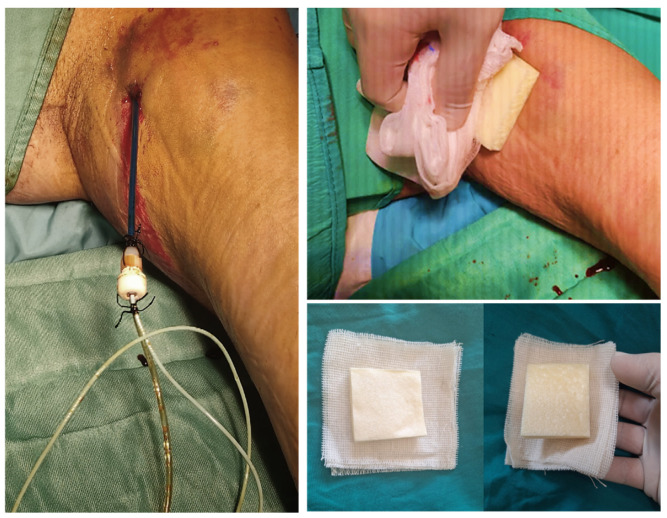
Axiostat application on the axillar arterial access site.

Once the procedure is finished, the skin around the vascular access site and the operator’s gloves are cleaned. The operator applies proximal pressure to the arterial access site and removes the introducer sheath. He then modulates the proximal pressure to verify the absence of bleeding from the access site (performing a blood wash to assess for arterial occlusions) and then initiates manual three-finger compression by placing Axiostat in contact with the access site (Figure 6).

Manual compression is performed for 10 min for sheath sizes between 4 and 8 Fr, after which the surgeon inspects the access site to assess the presence of expanding hematoma or significant bleeding through the puncture site. If this occurs, manual compression is continued until adequate hemostasis is achieved. Subsequently, a secondary adhesive compression dressing (Tensoplast Sport 10 cm × 2.5 mt) is applied over the Axiostat patch for a period of 12 h as per local protocol, and the same site is inspected by ultrasound. The Axiostat hemostatic pad is removed by first removing the secondary dressing and then irrigating the patch with plenty of water or saline solution. In this way, it turns into a liquid gel that can be removed easily, without causing pain, removing the clot and causing further bleeding. A color-coded duplex ultrasound is repeated immediately before discharge from hospital, and patients are examined after 30 days to rule out any complications. If problems are reported at the access site, the patient is invited to return immediately to our outpatient clinic for further clinical and duplex ultrasound follow-up, as per protocol.

### 4.3. Outcomes and Follow-Up

The primary objective of this study was to evaluate the efficacy and safety of the Axiostat hemostatic dressing in facilitating manual compression closure of the axillary artery access site in patients undergoing endovascular procedures for the treatment of Leriche syndrome. The primary efficacy endpoint was the technical success of axillary artery hemostasis, defined as single removal of the compression dressing within 10 min of placement without bleeding requiring immediate recompression. The secondary objective of the study was to evaluate and compare the efficacy, safety and performance of Axiostat at 24 h and 30 days post-procedure. Primary technical success was defined by adequate hemostasis achieved within 10 min of manual compression assisted by the Axiostat hemostatic dressing. Secondary technical success was defined as adequate hemostasis after Axiostat-assisted manual compression and successful intraoperative arterial closure without evidence of stenosis, occlusion or persistent bleeding requiring further intervention. Clinical success was defined by the absence of bleeding-related complications and follow-up. Clinical evaluation was defined as the success rate of axillary artery hemostasis, the incidence of axillary artery thrombosis and the presence of hematomas and false aneurysms at 24 h and 30 days after the procedure. Doppler ultrasound was conducted systematically at 24 h. Complications were classified according to SVS (Society for Vascular Surgery clinical practice guidelines) [26]. Time to hemostasis was defined as the elapsed time (in minutes) between removal of the introducer sheath and observation of hemostasis. Time to ambulation was defined as the elapsed time (in minutes) between application of the pressure dressing and the time when the patient can ambulate and resume activities free from the adhesive compression dressing.

### 4.4. Hemostasis Evaluation

**Figure 7:**
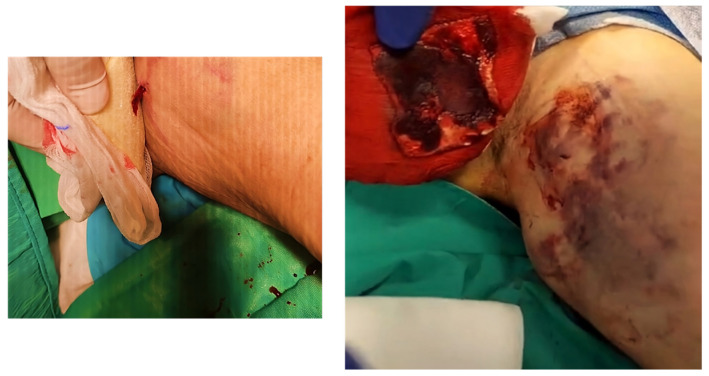
Hemostasis assessment after Axiostat removal.

The lesions were irrigated with saline in order to remove the dressing without affecting the blood clot. After removal of the Axiostat dressing, each lesion was qualitatively assessed for hemostasis at 6, 7 and 10 min after application using the validated bleeding scale (Figure 7).

### 4.5. Statistical Analysis

All statistical analyses for this study were performed on the recruited patient data using one-way ANOVA, with MATLAB R2023a^®^ (The MathWorks, Inc., Natick, MA, USA) software employed for this purpose [27]. For the Brown–Forsythe tests conducted, *p*-values of <0.05 and <0.001 were set as thresholds for statistical significance. Principal Component Analysis (PCA) is a chemometric statistical technique used for dimensionality reduction, data visualization and feature extraction. In our study, it was performed to uncover data patterns within the studied population. PCA decomposes the data matrix into orthogonal components, new descriptor variables called principal components (PCs). Each PC is a linear combination of the original variables, ordered to explain progressively smaller amounts of variance. The first PCs capture most of the data’s variability, enabling dimensional reduction and facilitating the interpretation of patterns and relationships among variables. Prior to analysis, the data were subjected to normalization and centering [28]. PCA results indicated that patients with higher BMI, diabetes and hyperlipidemia experienced prolonged hemostasis times, suggesting that metabolic factors influence the effectiveness of Axiostat. Chemometric analysis was conducted using the software package The Unscrambler X 10.4 (Camo Process As., Oslo, Norway).

## 5. Conclusions

Chitosan is one of the most studied biopolymers for surgical applications due to its biodegradability, biocompatibility and lack of toxicity. The versatility of this material is reflected in the wide variety of formulations and devices that have been made, highlighting, in particular, scaffolds, sponges and hydrogels. In the production of chitosan-based surgical materials, great importance has been attached to dressings for the control of hemostasis. In this regard, the objective of our study was to evaluate and test in vivo the ability of the chitosan-based Axiostat dressing as the device of choice in controlling bleeding at the access sites of vascular interventions. The results obtained show us that Axiostat is ≥99% effective as a hemostatic dressing with manual compression, thus ensuring hemostasis of the axillary arterial access site in patients undergoing endovascular procedure for the treatment of Leriche syndrome that are under a therapy regimen with anticoagulants and antiplatelets. The evaluation of hemostasis at the site of vascular access revealed that in this type of patient, where blood coagulation is impaired and the patient is predisposed to excessive bleeding, the application of Axiostat after endovascular intervention promoted platelet plug formation within 5 min. The minor failures that occurred in the patients studied were related to complications arising from endovascular procedures or errors on the part of health care providers; thus, they were not strictly related to the performance of the dressing. In this case, it can be said that this dressing is safe and effective in its in vivo application for the management of bleeding wounds in patients undergoing anticoagulant and antiplatelet therapy who undergo endovascular procedures at the axillary level. However, as our study is a single-center study, it is necessary, through future research, to include multicenter randomized clinical trials to validate these results in different patient populations and to explore alternative formulations of chitosan for greater efficacy.

## Figures and Tables

**Table 1:** Population data.

VARIABLES	ALL PATIENTS (N = 60)
Ages (Years)	67 ± 2.8 (67.4)
Sex (%M/%F)	40% M/60 F%
BMI *	27 ± 0.7 (27.1)
aPTT ** (S)	33 ± 0.8 (33.2)
Platelet Count (×10^6^/Ml)	330 ± 8.7 (330.6)
Diabetes Mellitus	40 (66%)
Coronary Artery Disease	8 (13%)
Congestive Heart Failure	11 (18%)
Cerebrovascular Disease	2 (3%)
Smoking History	45 (75%)
Hypertension	33 (56%)
Hyperlipidemia	37 (62%)
Chronic Renal Failure (Egfr < 60 Ml/Min)	6 (10%)

* Body mass index; ** activated partial thromboplastin time.

**Table 2:** Patient drug regimen.

VARIABLES	ALL PATIENTS (N = 60)
Mono-Antiaggregant Therapy	15 (25%)
Dual-Antiaggregant Therapy	33 (55%)
Anticoagulant Therapy	9 (15%)
Anticoagulant + Antiaggregant Therapy	4 (7%)

**Table 3:** Evaluation of outcome and follow-up. Significance was assessed by one-way ANOVA, which shows that the data collected during the experiments are statistically significant (*p* ≤ 0.036).

OUTCOMES	MONO-ANTIAGGREGANT THERAPY	DUAL-ANTIAGGREGANT THERAPY	ANTICOAGULANT THERAPY	ANTIAGGREGANT + ANTICOAGULANT THERAPY
PRIMARY SUCCES (%)	100	100	98	95.5
SECONDARY TECHINCAL SUCCES (%)	100	100	100	100
CLINICAL SUCCES (%)	100	100	98	96
SURGICAL SUCCES (%)	100	100	100	100
AMBULATION AFTER 24H (%)	100	100	100	100
RECOVERY OF FUNCTION (%)	100	100	98.8	96

**Table 4:** Evaluation of outcome and follow-up.

Outcomes	Mono-Antiaggregant Therapy (n° Control = 15)	Dual-Antiaggregant Therapy (n° Control = 33)	Anticoagulant Therapy (n° Control = 9)	Antiaggregant + Anticoagulant Therapy (n° Control = 4)
Hematoma	0	0	1	2
Pseudoaneurysm	0	5	2	0
Dissection	0	0	0	0
Arteriovenous fistula	0	0	0	0
Arterial thrombosis	1	0	0	1
Arterial stenosis	2	0	1	0
Hemorrhage	0	1	1	1
Infection	0	0	1	0
Neuropathy	0	2	0	0
Surgery	0	0	2	1
Drain removal after 24 h	0	0	2	1
Antibiotic therapy	0	0	2	3

**Table 5:** Hemostasis evaluation of Axiostat. Statistical significance was assessed by one-way ANOVA, which shows that the data collected during the experiments are statistically significant (*p* ≤ 0.0001).

All Patients (n = 60)	Hemostatic Success (%)	Hemostasis Time (min)	Hemostatic Insuccess (%)
Mono-Antiaggregant Therapy	100	5	0
Dual-Antiaggregant Therapy	100	6	0
Anticoagulant Therapy	99.5	5	0
Antiaggregant + Anticoagulant Therapy	98.2	7	1.8

## Data Availability

Data is contained in the paper.
